# Molecular ecology of highest priority critically important antibiotic resistant *Escherichia coli* from mammals housed at an urban zoo

**DOI:** 10.1093/jac/dkad148

**Published:** 2023-05-30

**Authors:** Jordan E Sealey, Richard Saunders, Teresa Horspool, Michelle G Barrows, Matthew B Avison

**Affiliations:** University of Bristol School of Cellular and Molecular Medicine, Biomedical Sciences Building, University Walk, Bristol BS8 1TD, UK; Bristol Zoological Society, Bristol Zoo Gardens, Clifton, Bristol, BS8 3HA, UK; Bristol Zoological Society, Bristol Zoo Gardens, Clifton, Bristol, BS8 3HA, UK; Bristol Zoological Society, Bristol Zoo Gardens, Clifton, Bristol, BS8 3HA, UK; University of Bristol School of Cellular and Molecular Medicine, Biomedical Sciences Building, University Walk, Bristol BS8 1TD, UK

## Abstract

**Objectives:**

Zoos are environments where species of highly valued animals are kept largely separated from others and the wider world. We report the molecular ecology of critically important antibiotic resistant (ABR) *Escherichia coli* carried by 28 mammalian species housed in a zoo located in an urban residential district.

**Methods:**

Over 3 months we collected 167 faecal samples from captive mammals and processed for *E. coli* resistant to third-generation cephalosporins (3GC-R) and fluoroquinolones (FQ-R). Isolates were sequenced using Illumina.

**Results:**

We identified high rates of faecal sample-level positivity, with 50%, 57% and 36% of mammalian species excreting 3GC-R, FQ-R or dual 3GC-R/FQ-R *E. coli*, respectively. Isolates represented multiple ST and ABR mechanisms; CTX-M-15 and CMY-2 dominated for 3GC-R, and target-site mutation caused 75% of FQ-R. We identified multiple examples of ABR *E. coli* transmission between mammalian species in separate enclosures, and a variant of the epidemic plasmid pCT within the zoo. There was no evidence for ABR *E. coli* leaving the zoo, based on comparative analysis with *E. coli* from humans, cattle and dogs isolated from the 50 × 50 km region in which the zoo is located. Amoxicillin/clavulanate was the most widely used antibiotic in the zoo, and we identified four widely disseminated amoxicillin/clavulanate resistance mechanisms, including a previously unreported inhibitor-resistant TEM, and the carbapenemase OXA-181.

**Conclusions:**

We conclude that the zoo studied here is a ‘melting pot’ for the selection and circulation of 3GC-R and FQ-R *E. coli*, but these circulating *E. coli* appear captive within the zoo.

## Introduction

Studies on antibiotic resistance (ABR) in zoo animals are rare and predominantly phenotypic,^1^ with one including MLST^2^ and two identifying ABR genes through PCR.^3,4^ Characterization of ABR bacteria is useful when considering selection and transmission of ABR, including zoonotic transmission.^5^ Our previous work has investigated One-Health transmission of ABR *Escherichia coli* within a 50 × 50 km study area. Focusing on third-generation cephalosporin resistance (3GC-R) and fluoroquinolone resistance (FQ-R), we have sequenced *E. coli* isolates from dairy cattle, dogs and humans.^6–12^

Within our study area, in an urban residential district, is a zoo. It is the world’s oldest provincial zoo, having been open since 1836. The aim of the study reported here was to investigate the molecular ecology of 3GC-R and FQ-R *E. coli* excreted by mammals within this zoo, to compare them with those from humans, cattle and dogs within our study area, and to investigate ABR selection drivers within the zoo.

## Materials and methods

### Study population

Random sampling of pooled faeces from animal enclosures at Bristol Zoological Gardens was performed by keepers during routine enclosure cleaning at three monthly timepoints between October and December 2020. Three replicates were taken to represent each enclosure at each timepoint, and samples were stored at 4°C and processed within 48 h. Each faecal sample was identified by keepers as having come from 1 of 28 mammalian species (Table S1, available as Supplementary data at *JAC* Online). Faeces from animals isolated due to poor health were not collected, so some enclosures were sampled on fewer than three occasions. Animals were not observed and there were no changes to routine husbandry practices or to the personnel entering each enclosure.

### Laboratory and genomics analysis

Replicate samples from the same enclosure and timepoint were pooled and weighed. Sample processing and selection of ABR *E. coli* was as described previously,^12^ using cefotaxime (2 mg/L) or ciprofloxacin (0.5 mg/L). Up to three isolates per selective agent per sample were analysed using three multiplex PCR assays: one to identify mobile β-lactamase genes,^6^ one to detect various *bla*_CTX-M_ groups,^6^ and one to detect plasmid-mediated quinolone resistance (PMQR) genes.^10^ One isolate per PCR profile, per selective agent, per sample collection timepoint, per mammalian species was selected as representative for WGS, performed by MicrobesNG and analysed for ABR and phylogeny as previously described.^10,12^ Quality control data are provided in Table S2. *E. coli* Fis2-e4 (NCBI accession CP041992.1) was used as the reference. Disc susceptibility testing and MIC assays were performed and interpreted according to CLSI guidelines.^13–15^

## Results and discussion

### Overview of 3GC-R and FQ-R E. coli

One hundred and sixty-seven faecal samples were collected from the enclosures of 28 mammalian species across three monthly timepoints. Not all animals could be sampled at each timepoint. 3GC-R or FQ-R *E. coli* were found in at least one sample from at least one timepoint in 14 (50%) and 16 (57%) animal species, respectively. Ten (36%) species excreted both 3GC-R and FQ-R *E. coli*, and eight (29%) did not excrete *E. coli* resistant to either (Table S1).

At sample level, 33% were positive for 3GC-R and 33% for FQ-R *E. coli*. This contrasted with our analysis of faecal samples from dairy farms (9.3% positive for 3GC-R; 6.3% for FQ-R; *n* = 4145 samples)^9^ and dogs (8.5% 3GC-R; 9.5% FQ-R; *n* = 600 dogs)^12^ and from human urine (5.8% 3GC-R; 9.9% FQ-R; *n* = 35 274 samples)^6^ collected within the 50 × 50 km study area including the zoo.

One hundred and thirty-one resistant *E. coli* isolates were analysed by PCR to identify mobile 3GC-R and PMQR genes, and one isolate per PCR profile per mammalian species per sample visit was selected for WGS, making 51 isolates in total. Eleven *E. coli* STs were identified through WGS of 26 3GC-R *E. coli*, with ST1722 the most common (Table S3). 3GC-R was due to *bla*_CTX-M-15_ (58% of isolates), *bla*_CMY-2_ (35%) or *bla*_CTX-M-14_ (8%). In total, there were 14 ST/ABR gene combinations (Table S3). 3GC-R was predominantly plasmid mediated (Table S4). An identical (based on read mapping) ∼90 kb IncI1 *bla*_CTX-M-15_ plasmid (assembled as a single contig) was found in 42% of sequenced 3GC-R isolates. This plasmid matched at 98% coverage with 99.7% identity with pJIE139 (accession: EU418926.1) carried by an Australian human urinary *E. coli*.^16^

A second IncI plasmid, being ∼94 kb and carrying *bla*_CMY-2_, was found in six *E. coli* STs, comprising 35% of isolates and 25% of sampled mammalian species (Table S4). Read mapping against its closest match, p92 (accession number: CP023376) from dogs in the UK,^17^ identified 70 SNP differences plus five insertions/deletions. There were three variants of this plasmid in the zoo, each differing by one SNP or insertion.

Among 25 sequenced FQ-R isolates, 14 ST/ABR gene combinations were identified (Table S5). Most FQ-R was caused by quinolone resistance-determining region (QRDR) mutation, with PMQR genes present in two STs carrying *qnrS1* and one carrying *qnrS2* plus *aac(6′)-lb-cr* (Table S5). In all cases, *aac(6′)-Ib-cr* was immediately upstream of *bla*_OXA-1_, with *qnrS2* also being adjacent, encoded on a novel IncFIB plasmid most similar to p14EC033f (accession number: CP024153.1).

### Phylogenetic analysis

**Figure 1.**
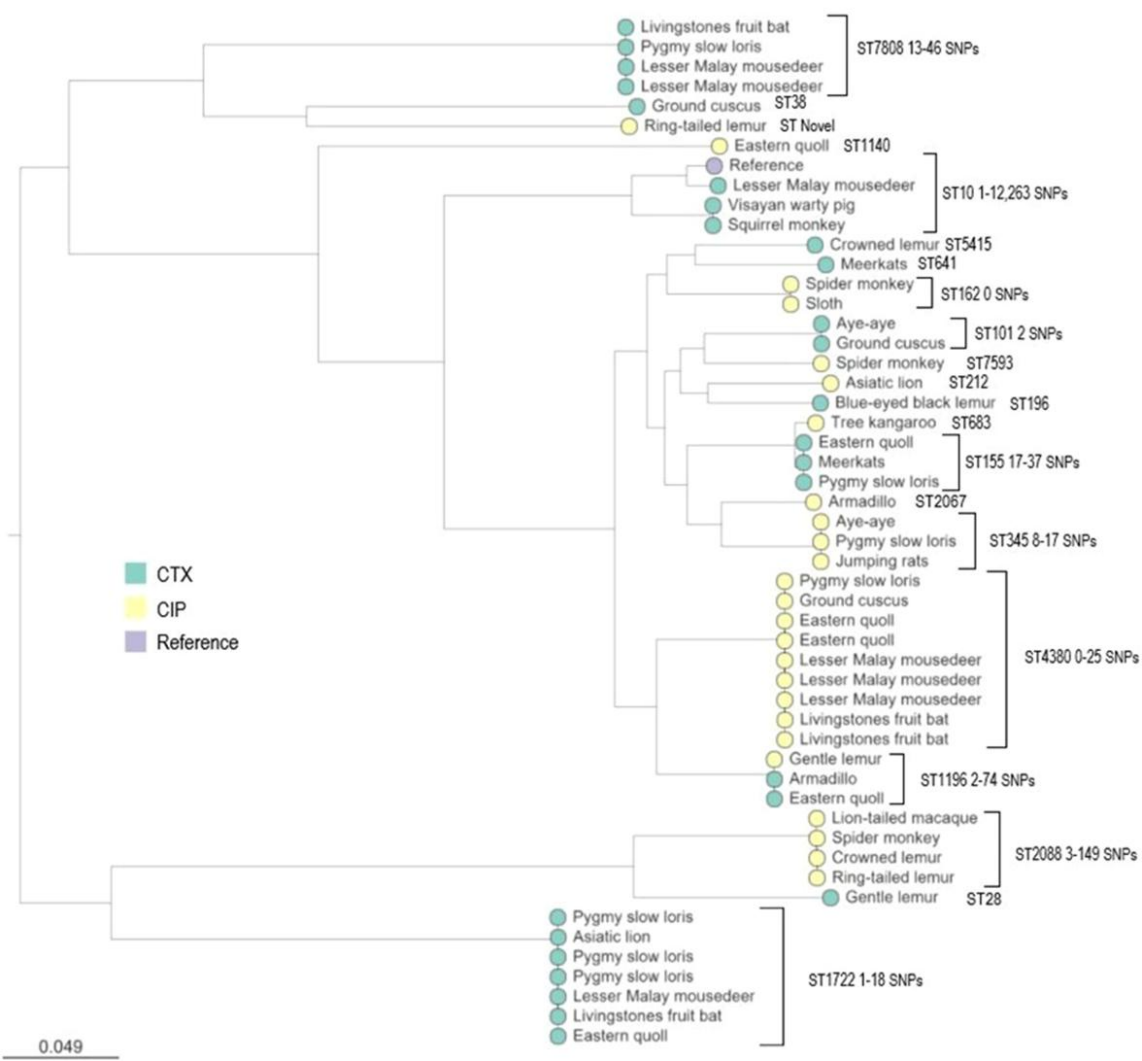
Core genome phylogenetic analysis of 3GC-R and FQ-R *E. coli* from captive mammals. Nodes of isolates are labelled with mammal species origin; ST and range of SNPs (bp) between isolates of the same ST are noted. Colour indicates selective medium used to isolate each (see key). ST1196 isolates selected on CTX are 3GC-R and FQ-R. Otherwise, an isolate’s 3GC-R/FQ-R phenotype reflects selection. This figure appears in colour in the online version of *JAC* and in black and white in the print version of *JAC*.

Figure 1 shows a mid-rooted phylogenetic tree based on core genome alignment of all 51 sequenced zoo isolates. In most cases SNP distances across groups of isolates from the same ST were <15, even across multiple mammalian species, indicative of ongoing circulation of isolates between animals within the zoo. However, none of the ST/ABR gene combinations found in the zoo (Tables S3 and S5) were found among 609 3GC-R and/or FQ-R *E. coli* from humans, cattle or dogs in the same study area.^6–8,10,12^

One example of potential ABR plasmid dissemination into the zoo was a *bla*_CTX-M-14_ plasmid (Table S4), with only five SNP differences from the IncK plasmid pCT.^18^ pCT is classed as an epidemic plasmid for *bla*_CTX-M-14_, being responsible for 30% of *bla*_CTX-M-14_ horizontal transfer in *E. coli* from humans, cattle and poultry in the UK,^19^ and pCT has been found in our study region in *E. coli* from humans and cattle but not dogs.^6,8,12^

### β-Lactam/β-lactamase inhibitor resistance

Isolates representative of 3GC-R and FQ-R ST/ABR gene combinations were subjected to disc susceptibility testing (Table S6). Amoxicillin/clavulanate non-susceptibility was seen in all isolates producing CMY-2 or OXA-1, as expected.^20^ All CTX-M producers, including those carrying *bla*_TEM-1_, were amoxicillin/clavulanate susceptible, except for one ST1722 combination (Table S6). This was found to carry a *bla*_TEM-33-like_ gene, which is not recorded on any public sequence repository. Like TEM-33, this novel TEM has a leucine at position 69, which has been associated with reduced inhibitor binding.^21^ The only difference from TEM-33 is a Trp133Cys substitution. Additionally, a Pa −10 promoter mutation was found upstream of this *bla*_TEM-33-like_ gene, where all *bla*_TEM-1_ genes found at the zoo had WT promoters (Figure S1). Pa −10 promoter mutations confer amoxicillin/clavulanate resistance because they cause TEM overproduction.^20^ We confirmed that the *bla*_TEM-33-like_ gene is located on a p0111-type plasmid sharing 92% identity with pA16EC0618-5 (accession: CP088846.1). All sequenced *bla*_TEM-33-like_-containing plasmids were identical, confirming widespread circulation in the zoo.

All CMY-2 β-lactamase producers were piperacillin/tazobactam susceptible (Table S6) except for ST155 isolates carrying the carbapenemase gene *bla*_OXA-181_, also found in one FQ-R isolate (Table S6). The ST155 and FQ-R isolates were ertapenem (MICs 2 mg/L and 4 mg/L, respectively) and piperacillin/tazobactam (MICs 128/4 mg/L and 256/4 mg/L) resistant. In the zoo, *bla*_OXA-181_ was only found on an identical ∼51 kb IncX3 plasmid (Table S6) having 100% coverage and 99.99% identity to plasmid C013421 (accession number CP068328.1) from a human *E. coli* in the Netherlands.^22^

### Antibiotic usage

Analysis of electronic medical records allowed cumulative dispensing of antibiotics for treatment of mammals at the zoo to be reported (Table 1). The highest usage (by weight) was of ciprofloxacin, amoxicillin, and amoxicillin/clavulanate. In terms of number of doses administered, however, and the breadth of species receiving these doses, amoxicillin/clavulanate was by far the most widely used, comprising 51.8% of all antibiotic doses (Table 1). It may well be, therefore, that high amoxicillin/clavulanate usage is a driver of the extensive array of amoxicillin/clavulanate resistance mechanisms seen among 3GC-R and/or FQ-R *E. coli* in the zoo. Importantly, this can co-select for resistance to piperacillin/tazobactam (CMY, OXA-1/-181), 3GCs (CMY), FQs [AAC(6′)-Ib-cr, QnrS, encoded alongside amoxicillin/clavulanate resistance genes] and ertapenem (OXA-181).

**Table 1.** Antibiotics dispensed for treatment of mammals at Bristol Zoo, September 2016 to September 2021

Antibiotic	Amount (mg) dispensed	Defined daily doses	No. of species treated
Ciprofloxacin	230 425	316	3
Marbofloxacin	24 786 (10 387)	1026 (726)	13 (9)
Enrofloxacin	4206 (859)	335 (30)	13 (8)
Amoxicillin/clavulanate	258 357 (206 297)	3212 (2549)	36 (23)
Amoxicillin	214 295 (195 183)	228 (195)	27 (18)
Ceftazidime	4000 (1000)	7 (1)	2 (1)
Ceftiofur	654	138	2
Cefovecin	964 (804)	7 (5)	4 (3)
Doxycycline	976 (764)	166 (90)	4 (2)
Tulathromycin	43 (35)	8 (7)	2 (1)
Trimethoprim/sulfamethoxazole	143 985 (135 372)	758 (617)	17 (13)

Not all the mammalian species present at the zoo and receiving treatment over the 5 year period of this medicines usage survey were available for faecal sampling in this study. Numbers in brackets relate to the 28 mammalian species sampled in this study, if the total includes additional species not sampled.

Despite being in the heart of a residential part of the city, we found no evidence of ‘escape’ of the novel TEM, OXA-181 or OXA-1 plasmids identified above, or any other ST/ABR gene combination found in the zoo into *E. coli* colonizing dogs or cattle, or infecting humans in the same city, or surrounding region. ABR was found to be readily circulating within the zoo, however, even between animals in enclosures separated by some distance. This could be due to shared food or food preparation space, or because keepers or their equipment move between enclosures. However, wider sampling of people, equipment and the environment would be required to investigate further.

## Supplementary Material

dkad148_Supplementary_Data

## Supplementary data

Figure S1 and Tables S1–S6 are available as Supplementary data at *JAC* Online.
