# Supplementary material for: Molecular ecology of highest priority critically important antibiotic resistant Escherichia coli from mammals housed at an urban zoo
Source: J Antimicrob Chemother. 2023 May 30;78(7):1667–71. doi: 10.1093/jac/dkad148 (PMC10320166; doi:10.1093/jac/dkad148)

**Table S1. Excretion of 3GC-R and FQ-R *E. coli* in captive mammals**

|  |  | Positive or Negative for *E. coli* | | |
| --- | --- | --- | --- | --- |
| Species | Common name | No antibiotic | 3GC-R | FQ-R |
| *Hylobates agilis* | Agile gibbon | + | - | - |
| *Euphractus sexcinctus* | Six-banded armadillo | + | + | + |
| *Panthera leo persica* | Asiatic lion | + | + | + |
| *Daubentonia madagascariensis* | Aye-aye | + | + | + |
| *Bettongia penicillata* | Bettong | + | - | - |
| *Eulemur flavifrons* | Blue-eyed black lemur | + | + | - |
| *Eulemur coronatus* | Crowned lemur | + | + | + |
| *Dasyurus viverrinus* | Eastern quoll | + | + | + |
| *Hapalemur alaotrensis* | Gentle lemur | + | + | + |
| *Leontopithecus chrysomelas* | Golden-headed lion tamarin | + | - | - |
| *Gorilla gorilla gorilla* | Western lowland gorilla | + | - | - |
| *Phalanger gymnotis* | Ground cuscus | + | + | + |
| *Hypogeomys antimena* | Malagasy Jumping rat | + | - | + |
| *Tragulus javanicus* | Lesser Malay mousedeer | + | + | + |
| *Macaca silenus* | Lion tailed macaque | + | - | + |
| *Pteropus livingstonii* | Livingstone's fruit bat | + | + | + |
| *Suricata suricatta* | Meerkats | + | + | - |
| *Dolichotis patagonum* | Patagonian mara | - | - | - |
| *Choeropsis liberiensis* | Pygmy hippo | + | - | - |
| *Nycticebus pygmaeus* | Pygmy slow loris | + | + | + |
| *Ailurus fulgens* | Red panda | + | - | - |
| *Lemur catta* | Ring-tailed lemur | + | - | + |
| *Choloepus didactylus* | Two-toed Sloth | + | - | + |
| *Ateles hybridus* | Brown spider monkey | + | - | + |
| *Saimiri sciureus* | Squirrel monkey | + | + | - |
| *Dendrolagus goodfellowi* | Goodfellow’s tree kangaroo | + | - | + |
| *Sus cebifrons* | Visayan warty pig | + | + | - |
| *Cynictis penicillata* | Yellow mongoose | + | - | - |

**Table S2. Quality control data for WGS**

| **Sample id** | **# contigs (>= 0 bp)** | **# contigs (>= 1000 bp)** | **Total length (>= 0 bp)** | **Total length (>= 1000 bp)** | **# contigs** | **Largest contig** | **Total length** | **GC (%)** | **N50** | **N75** | **L50** | **L75** | **# N's per 100 kbp** |
| --- | --- | --- | --- | --- | --- | --- | --- | --- | --- | --- | --- | --- | --- |
| 34427_Z1011CTXR | 199 | 73 | 5312310 | 5272199 | 93 | 514553 | 5286523 | 50.62 | 164120 | 118653 | 9 | 18 | 0.00 |
| 34428_Z1041CTXR | 107 | 35 | 5112104 | 5091991 | 40 | 712994 | 5095556 | 50.51 | 496286 | 239202 | 5 | 8 | 0.00 |
| 34429_Z1051CTXR | 324 | 57 | 5056432 | 4965555 | 106 | 478927 | 4998644 | 50.28 | 236031 | 152925 | 8 | 15 | 0.00 |
| 34431_Z1071CTXR | 73 | 31 | 4871342 | 4859715 | 33 | 892189 | 4861222 | 50.70 | 493531 | 223737 | 4 | 8 | 0.00 |
| 34433_Z1101CTXR | 147 | 70 | 5096203 | 5073460 | 78 | 659767 | 5079773 | 50.75 | 150541 | 76478 | 11 | 22 | 0.00 |
| 34434_Z1131CTXR | 195 | 53 | 4781003 | 4723399 | 83 | 432127 | 4744308 | 50.71 | 213940 | 119731 | 8 | 17 | 0.00 |
| 34435_Z1141CTXR | 92 | 41 | 5012163 | 4999955 | 42 | 707497 | 5000839 | 50.52 | 317615 | 145503 | 5 | 11 | 0.00 |
| 34436_Z1151CTXR | 59 | 29 | 4868492 | 4861217 | 30 | 703954 | 4861903 | 50.70 | 535750 | 223855 | 4 | 8 | 0.00 |
| 34437_Z1171CTXR | 189 | 69 | 5336554 | 5293408 | 97 | 567647 | 5312059 | 50.59 | 217890 | 118653 | 8 | 17 | 0.00 |
| 34438_Z1231CTXR | 263 | 81 | 4983974 | 4904099 | 137 | 503697 | 4939475 | 50.62 | 138611 | 83838 | 10 | 22 | 0.00 |
| 34439_Z1011CIPR | 89 | 33 | 4990741 | 4975755 | 36 | 1178949 | 4978017 | 50.75 | 312826 | 218166 | 4 | 9 | 0.00 |
| 34441_Z1041CIPR | 93 | 32 | 4709011 | 4692557 | 35 | 1178949 | 4694819 | 50.97 | 312826 | 218166 | 4 | 9 | 0.00 |
| 34442_Z1071CIPR | 137 | 40 | 4771671 | 4738647 | 56 | 1178949 | 4748693 | 50.89 | 267183 | 159748 | 5 | 10 | 0.00 |
| 34443_Z1141CIPR | 144 | 33 | 4731660 | 4693674 | 42 | 1178949 | 4700240 | 50.96 | 269776 | 159748 | 5 | 10 | 0.00 |
| 34445_Z1161CIPR | 174 | 49 | 4956670 | 4915001 | 60 | 614637 | 4923964 | 50.66 | 277026 | 144179 | 6 | 12 | 0.00 |
| 34446_Z1171CIPR | 166 | 63 | 5143409 | 5108970 | 78 | 379043 | 5119218 | 50.58 | 195119 | 97799 | 10 | 19 | 0.00 |
| 34447_Z1241CIPR | 353 | 61 | 5078971 | 4940322 | 159 | 885280 | 5007759 | 50.60 | 277026 | 166612 | 5 | 10 | 0.00 |
| 34448_Z1242CIPR | 97 | 53 | 4966800 | 4948770 | 65 | 624795 | 4957291 | 50.50 | 289269 | 189834 | 6 | 11 | 0.00 |
| 39671_Z1321CIPR | 118 | 59 | 4974140 | 4950318 | 73 | 636087 | 4960993 | 50.50 | 275128 | 189834 | 6 | 11 | 0.00 |
| 39672_Z1331CIPR | 70 | 30 | 4782534 | 4769913 | 35 | 636735 | 4773102 | 50.83 | 461648 | 274160 | 5 | 8 | 0.00 |
| 39673_Z1381CIPR | 95 | 32 | 4710940 | 4694508 | 34 | 1178949 | 4696111 | 50.97 | 312641 | 159953 | 4 | 10 | 0.00 |
| 39674_Z1471CIPR | 210 | 56 | 5091306 | 5035903 | 81 | 661465 | 5052148 | 50.69 | 348450 | 172782 | 6 | 11 | 0.00 |
| 40083_Z1491CTXR | 101 | 35 | 4581752 | 4561225 | 43 | 1146326 | 4566548 | 50.87 | 281317 | 263420 | 5 | 9 | 0.00 |
| 40084_Z1531CTXR | 77 | 35 | 4946341 | 4935655 | 37 | 706051 | 4936929 | 50.50 | 317615 | 159367 | 5 | 10 | 0.00 |
| 40085_Z1541CTXR | 118 | 46 | 5053770 | 5030768 | 56 | 705927 | 5037346 | 50.45 | 299361 | 145279 | 5 | 11 | 0.00 |
| 40086_Z1542CTXR | 1109 | 60 | 5109082 | 4915559 | 72 | 441827 | 4924191 | 50.79 | 257333 | 101025 | 8 | 15 | 0.00 |
| 40087_Z1571CTXR | 464 | 46 | 5139931 | 5057436 | 52 | 705722 | 5060920 | 50.54 | 288205 | 137118 | 6 | 13 | 0.02 |
| 40088_Z1711CTXR | 574 | 51 | 4986050 | 4883371 | 57 | 478735 | 4886898 | 50.43 | 217471 | 158988 | 8 | 15 | 0.00 |
| 40089_Z1721CTXR | 123 | 43 | 5083570 | 5059522 | 48 | 706050 | 5062667 | 50.53 | 318115 | 145503 | 5 | 11 | 0.00 |
| 40090_Z1722CTXR | 1770 | 65 | 5221023 | 4921048 | 74 | 628627 | 4926948 | 50.71 | 211175 | 93725 | 7 | 17 | 0.00 |
| 40091_Z1501CIPR | 128 | 51 | 4950342 | 4923553 | 58 | 405484 | 4928640 | 50.68 | 227490 | 136976 | 8 | 15 | 0.00 |
| 40092_Z1511CIPR | 740 | 397 | 6053109 | 5880300 | 527 | 280765 | 5967459 | 49.98 | 49745 | 21202 | 33 | 80 | 0.00 |
| 40093_Z1531CIPR | 107 | 34 | 4680987 | 4659284 | 38 | 1138006 | 4662427 | 50.94 | 312826 | 218166 | 4 | 8 | 0.00 |
| 40094_Z1541CIPR | 103 | 35 | 4678447 | 4657651 | 38 | 1178610 | 4660049 | 50.96 | 320243 | 218166 | 4 | 9 | 0.00 |
| 40095_Z1571CIPR | 157 | 46 | 4949175 | 4905291 | 58 | 404344 | 4913119 | 50.68 | 217363 | 160155 | 8 | 14 | 0.00 |
| 40096_Z1581CIPR | 117 | 32 | 4953912 | 4929839 | 40 | 931433 | 4936122 | 50.57 | 359829 | 200651 | 5 | 9 | 0.00 |
| 40098_Z1592CIPR | 103 | 38 | 4943668 | 4921846 | 47 | 526164 | 4928759 | 50.67 | 295789 | 186700 | 7 | 12 | 0.00 |
| 40100_Z1721CIPR | 107 | 29 | 4710147 | 4689786 | 34 | 1522837 | 4693673 | 50.97 | 312826 | 218166 | 3 | 8 | 0.00 |
| 40101_Z1722CIPR | 133 | 73 | 5251746 | 5232532 | 78 | 391638 | 5236459 | 50.62 | 176650 | 93368 | 12 | 22 | 0.00 |
| 40102_Z1111CIPR | 124 | 42 | 4963264 | 4936947 | 45 | 1150421 | 4939318 | 50.78 | 397164 | 133545 | 4 | 9 | 0.00 |
| 40162_Z1271CTXR | 522 | 132 | 5786517 | 5628568 | 232 | 705722 | 5698336 | 49.03 | 316474 | 159367 | 6 | 12 | 0.00 |
| 40163_Z1281CTXR | 509 | 41 | 5020542 | 4875859 | 93 | 892934 | 4910438 | 50.74 | 383130 | 224082 | 4 | 8 | 0.00 |
| 40164_Z1311CTXR | 323 | 75 | 5133180 | 5043713 | 112 | 663437 | 5068825 | 50.51 | 182598 | 97810 | 8 | 18 | 0.00 |
| 40165_Z1371CTXR | 140 | 43 | 5091086 | 5058019 | 48 | 706051 | 5061594 | 50.54 | 318115 | 155179 | 5 | 11 | 0.00 |
| 40166_Z1381CTXR | 203 | 48 | 4935209 | 4891430 | 54 | 617064 | 4896386 | 50.63 | 238290 | 121379 | 7 | 15 | 0.00 |
| 40167_Z1391CTXR | 996 | 42 | 5001595 | 4827186 | 51 | 628627 | 4833270 | 50.78 | 320463 | 162025 | 6 | 11 | 0.00 |
| 40168_Z1421CTXR | 178 | 40 | 4948165 | 4917828 | 45 | 1498759 | 4921255 | 50.74 | 326349 | 162025 | 4 | 9 | 0.00 |
| 40170_Z1471CTXR | 89 | 39 | 5065506 | 5047951 | 48 | 766477 | 5053572 | 50.50 | 396687 | 156867 | 5 | 9 | 0.00 |
| 40171_Z1271CIPR | 583 | 66 | 5127309 | 5011875 | 83 | 311348 | 5024240 | 50.71 | 182995 | 106402 | 11 | 21 | 0.00 |
| 40172_Z1281CIPR | 99 | 33 | 4677302 | 4658565 | 36 | 1178610 | 4661154 | 50.95 | 312641 | 218166 | 4 | 9 | 0.00 |
| 40173_Z1311CIPR | 108 | 52 | 4972618 | 4948662 | 61 | 635646 | 4955475 | 50.50 | 275128 | 189831 | 6 | 11 | 0.00 |

**Table S3. ST and ABR gene profiles identified in 3GC-R *E. coli* isolates from captive mammals.**

| **ST** | **3GC-R genes** | **Other ABR genes** | **Species** |
| --- | --- | --- | --- |
| ST1722 | *bla*_CTX-M-15_ | *bla*_TEM-33-like_ | Asiatic lion, eastern quoll, lesser Malay mousedeer, pygmy slow loris^+^. |
| ST1722 | *bla*_CTX-M-15_ |  | Livingstone’s fruit bat. |
| ST7808 | *bla*_CTX-M-15_ |  | Lesser Malay mousedeer^+^, Livingstone’s fruit bat, pygmy slow loris. |
| ST10 | *bla*_CTX-M-15_ | *bla*_TEM-1_, *dfrA*, *strA*, *strB*, *sul*2, *tet*(A), *qnrS1* | Squirrel monkey, Visayan warty pig. |
| ST10 | *bla*_CTX-M-15_ | *bla*_TEM-1_, *dfrA*, *strA*, *strB*, *sul*2, *tet*(A) | Lesser Malay mousedeer. |
| ST196 | *bla*_CTX-M-15_ | *qnrS1* | Blue-eyed black lemur. |
| ST1196* | *bla*_CTX-M-14_ | *tet*(B) | Eastern quoll. |
| ST1196* | *bla*_CTX-M-14_ |  | Armadillo. |
| ST101 | *bla*_CMY-2_ | *sul*2, *tet*(B) | Aye-aye, ground cuscus. |
| ST155 | *bla*_CMY-2_ | *bla*_OXA-181_, *qnrS1* | Eastern quoll, meerkats, pygmy slow loris. |
| ST28 | *bla*_CMY-2_ |  | Gentle lemur. |
| ST38 | *bla*_CMY-2_ |  | Ground cuscus. |
| ST641 | *bla*_CMY-2_ |  | Meerkats. |
| ST5415 | *bla*_CMY-2_ |  | Crowned lemur. |
| ^+^Found in the species over more than one time point  *Isolates have 4 QRDR mutations giving FQ-R. | | | |

**Table S4. 3GC-R plasmids identified in *E. coli* from zoo mammals.**

| **Plasmid** | **Replicon Type** | **3GC-R genes** | **Other ABR genes** | **ST** | **Species** |
| --- | --- | --- | --- | --- | --- |
| BZP1 | IncI1-I(alpha) (pMLST unknown) | *bla*_CTX-M-15_ |  | ST10, ST1722, ST7808 | Asiatic lion, eastern quoll, lesser Malay mousedeer, Livingstone’s fruit bat, pygmy slow loris. |
| BZP2 | Untypable | *bla*_CTX-M-15_ | *bla*_TEM-1_, *dfrA*, *strA*, *strB*, *sul*2, *tet*(A), *qnrS1* | ST10 | Squirrel monkey, Visayan warty pig. |
|  |  |  |  |  |  |
| BZP3 | pCT, IncK | *bla*_CTX-M-14_ |  | ST1196 | Eastern quoll, armadillo. |
| BZP4 | IncI1-I(alpha) (pMLST 2, CC-2) | *bla*_CMY-2_ |  | ST28, ST38, ST101, ST155, ST641, ST5415 | Aye-aye, crowned lemur, Eastern quoll, gentle lemur, ground cuscus, meerkats, pygmy slow loris. |
|  |  |  |  |  |  |

**Table S5. ST and ABR gene profiles identified in FQ-R *E. coli* isolates from captive mammals.**

| **ST** | **FQ-R genes/ mutations** | **Other ABR genes** | **Species** |
| --- | --- | --- | --- |
| ST4380 | QRDR**** | *dfrA1*, *strA*, *strB*, *sul2*, *tet*(A) | Eastern quoll^+^, ground cuscus, lesser Malay mousedeer^+^, Livingstone’s fruit bat^+^, pygmy slow loris. |
| ST2088 | *aac(6’)Ib-cr*, *qnrS2* | *bla*_OXA-1_, *bla*_TEM-1_, *catB3*, *sul*3, *tet*(A) | Ring-tailed lemur, spider monkey |
| ST2088 | *aac(6’)Ib-cr*, *qnrS2* | *aadA2*, *bla*_OXA-1_, *bla*_TEM-1_, *catB3*, *dfrA12*, *sul3*, *tet*(A) | Lion-tailed macaque |
| ST2088 | *aac(6’)Ib-cr*, *qnrS2* | *bla*_OXA-1_, *bla*_TEM-1_, *catB3*, *sul*3 | Crowned lemur |
| ST345 | QRDR*** | *aadA2*, *dfrA12*, *sul*1, *tet*(A) | Aye-aye, jumping rats, pygmy slow loris |
| ST162 | QRDR*** | *dfrA1* | Spider monkey |
| ST162 | QRDR*** |  | Sloth |
| ST212 | QRDR*** | *aadA1*, *bla*_TEM-1_, *dfrA1*, *sul1*, *sul2*, *strA*, *strB*, *tet*(A) | Asiatic lion |
| ST683 | *qnrS1* | *dfrA14*, *sul2*, *strA* | Tree kangaroo |
| ST1140 | QRDR*** | *aadA1*, *aadA2*, *bla*_TEM-1_, *cmlA1*, *dfrA1*, *sul3*, *tet*(A) | Eastern quoll |
| ST1196 | QRDR**** | *aadA1*, *aadA2*, *bla*_TEM-1_, *cmlA1*, *dfrA12*, *floR*, *mph*(A), *sul3*, *tet*(A), *tet*(M) | Gentle lemur |
| ST2067 | QRDR*** |  | Armadillo |
| ST7593 | QRDR*** | *bla*_TEM-1_, *dfrA14*, *sul2*, *strA*, *strB*, *tet*(A) | Spider monkey |
| ST novel | *qnrS1*, QRDR* | *bla*_OXA-181_, *bla*_TEM-33-like_ | Ring-tailed lemur |
| ^+^Found in species over more than one time point.  QRDR mutations:  *****gyrA*(Ser83Leu), *gyrA*(Asp87Asn), *parC*(Ser80Ile), *parE*(Ser458Ala); ****gyrA*(Ser83Leu), *gyrA*(Asp87Asn), *parC*(Ser80Ile);  * *gyrA*(Ser83Leu). | | | |

**Table S6. Antimicrobial susceptibility testing of 3GC-R and FQ-R *E. coli* from captive mammals.**

|  | *E. coli* | Resistance genes | Antibiotic Resistance by disc susceptibility |
| --- | --- | --- | --- |
|  | ST28 | *bla*_CMY-2_ | **AMC**, ATM (I), CAZ, CTX, FOX |
|  | ST38 | *bla*_CMY-2_ | **AMC**, ATM (I), CTX, FOX |
|  | ST641 | *bla*_CMY-2_ | **AMC**, CAZ (I), CTX, FOX |
|  | ST5415 | *bla*_CMY-2_ | **AMC**, CTX, FOX |
|  | ST101 | *bla*_CMY-2_, *sul2*, *tet*(B) | **AMC**, ATM, CAZ, CTX, FOX, TE |
|  | ST155 | *bla*_CMY-2_, *bla*_OXA-181_, *qnrS1* | **AMC**, ATM (I), CAZ, CTX, FOX, **TZP**, CIP (I) |
|  | ST7808 | *bla*_CTX-M-15_ | ATM (I), CTX, FEP (I) |
|  | ST196 | *bla*_CTX-M-15_, *qnrS1* | ATM, CAZ (I), CTX, FEP, CIP (I) |
|  | ST1196 | *bla*_CTX-M-14_, *tet*(B), QRDR**** | CTX, FEP, CIP, TE |
|  | ST10 | *bla*_CTX-M-15_, *bla*_TEM-1_, *dfrA14*, *strA*, *strB*, *sul2*, *tet*(A), *qnrS1*. | ATM, CAZ (I), CTX, FEP, CIP (I), SXT, TE |
|  | ST1722 | *bla*_CTX-M-15_ | ATM (I), CTX, FEP (I) |
|  | ST1722 | *bla*_CTX-M-15_, *bla*_TEM-33-like_ | **AMC** (I), ATM, CTX, FEP (I), **TZP** (I) |
|  | ST4380 | *dfrA1*, *strA*, *strB*, *sul2*, *tet*(A), QRDR**** | CIP, SXT, TE |
|  | ST2088 | *aac(6’)-Ib-cr*, *aadA2*, *bla*_OXA-1_, *bla*_TEM-1_, *catB3*, *dfrA12*, *sul3*, *tet*(A), *qnrS2* | **AMC (I), TZP** (I), CIP, SXT, TE |
|  | ST345 | *aadA2*, *dfrA12*, *sul1*, *tet*(A), QRDR*** | CIP, SXT, TE |
|  | ST162 | dfrA1, QRDR*** | AMC (I), FEP (I), CIP, TE |
|  | ST212 | *aadA1*, *bla*_TEM-1_, *dfrA1*, *sul1*, *sul2*, *strA*, *strB*, *tet*(A), QRDR*** | CIP, SXT, TE |
|  | ST683 | *dfrA14*, *sul2*, *strA*, *qnrS1* | **TZP (I)**, CIP, SXT (I) |
|  | ST1140 | *aadA1*, *aadA2*, *dfrA1*, *bla*_TEM-1_, *cmlA1*, *sul3*, *tet*(A), QRDR*** | C, CIP, SXT, TE |
|  | ST1196 | *aadA1*, *aadA2*, *bla*_TEM-1_, *cmlA1*, *dfrA12*, *floR*, *mph*(A), *sul3*, *tet*(A), *tet*(M), QRDR**** | C, CIP, SXT, TE |
|  | ST2067 | QRDR*** | CIP |
|  | ST7593 | *bla*_TEM-1_, *dfrA14*, *sul2*, *strA*, *strB*, *tet*(A), QRDR*** | CIP, SXT, TE |
|  | ST unknown | *bla*_OXA-181_, *bla*_TEM-33-like_, *qnrS1*, QRDR* | **AMC**, **TZP**, CIP |
|  | (I) = Intermediate resistance in disc susceptibility testing. * symbolises number of QRDR mutations. | | |

**Figure S1**

**CLUSTAL OMEGA alignment of *bla*_TEM_ resistance gene upstream sequences from *E. coli* in zoo mammals.** (A) nine isolates carrying *bla*_TEM-1_ and (B) seven isolates carrying the *bla*_TEM-33-like_ gene (**Table S2**) showing a C-T promoter mutation at Pa -10 (highlighted in yellow) in the overlapping Pb-35/Pa-10 region (highlighted in green) increasing the consensus match from 2/6 to 3/6. The other two promoter regions: Pb and P3 were identical from these bacteria. Stars indicate identities across all isolates.


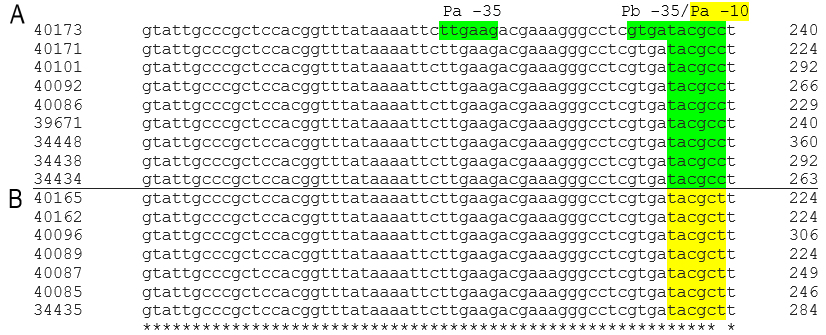

Supplement: dkad148_Supplementary_Data [file dkad148_supplementary_data.docx]
